# Selected Risk Factors and Pattern of Semen Abnormality in Male Partners of Infertile Couples in Eastern Nepal: A Descriptive Cross-sectional Study

**DOI:** 10.31729/jnma.4882

**Published:** 2020-09-30

**Authors:** Sita Pokhrel, Ashima Ghimire, Manisha Chhetry, Sabina Lamichane, Rupesh Kumar Shreewastav

**Affiliations:** 1 Department of Obstetrics and Gynecology, Nobei Medical College Teaching Hospital, Biratnagar, Nepal; 2 Department of Biochemistry, Nobel Medical College Teaching Hospital, Biratnagar, Nepal

**Keywords:** *infertility*, *risk factors*, *semen abnormality parameters*

## Abstract

**Introduction:**

Semen analysis is an initial basic step in evaluating and diagnosing male infertility. Multiple risks factors in combination or alone are responsible for abnormal semen parameters. The present study aimed to study certain risk factors and semen parameters of infertile male.

**Methods:**

It was a descriptive cross-sectional study. We consecutively enrolled 186 male partners of infertile couple who underwent certain risk factors evaluation and semen analysis according to WHO guideline.

**Results:**

Multiple risk factors were present like Gulf country migration, smoking, chemical exposure and heat exposure in infertile male partners. Forty six percent of our patients were gulf workers. Eleven percent patients had azoospermia, 27% had abnormal sperm morphology and 23% had <25% motile spermatozoa.

**Conclusions:**

Surprisingly 46% of our patients were Gulf country workers and abnormal semen analysis is very important factor for infertility. Large prospective studies need to be carried out involving Gulf migrant workers only.

## INTRODUCTION

In our male dominated society, the blame for infertility goes more to female partners and they suffer from domestic violence, economic deprivation, social neglect, separation and mental trauma.^1,2^ If different parameters of semen analysis are interpreted with clinical profile of the male partner it would give more information for proper future management.^3^

Functional competence of the spermatozoa may be associated with certain semen parameters.^4^ Different studies showed that causes of infertility is attributed to female in 1/3^rd^ of cases, male in 1/3^rd^ and unexplained in 1/3^rd^.^5^ Factors like diabetes mellitus, chronic medication and disease, psychological factors, chronic alcohol consumption of alcohol, smoking, adverse occupation, nutritional factors and infections play role.^6,7^

Therefore, we wanted to look into the patterns of semen parameters and certain risk factors associated with abnormality in male partners of infertile couple in our setting. It will help to understand the real pattern and parameters of investigation.

## METHODS

This was a descriptive cross-sectional study of the certain risk factors and seminal fluid indices of consecutively consenting male partners of infertile couples seen in the department of Obstetrics and Gynecology, Nobel Medical College Teaching Hospital, Biratnagar. The cases were male partner of a couple, who visited the department for infertility treatment in the period of 22^nd^ January 2017 to 21^st^ January 2018. The purpose of the study was explained to the couple, who agreed to be the part of the study was included. The study was carried out after getting the approval of Institutional Review Committee.

We recorded important risk factors and semen parameters in a structured proforma. Information related to risk factors and semen parameters were noted. The male partners were adequately counseled and given instructions on how to collect the semen samples. These instructions included abstinence from coitus for 3-5 days, washing their hands before starting masturbation, sample collection by masturbation only accepted and kept close to the body and delivered to the laboratory within 30 minutes of collection. Spilled samples were avoided. All samples were collected into sterile screw capped plastic universal containers. The semen samples were collected in a dedicated room with bed and other facilities to make them relax within the laboratory and analysed within one hour of collection.

The semen analysis was performed according to the methods and standard outlined by WHO guideline. The sample analysis was done by same laboratory scientist to avoid inter-personal variations. Semen analysis was done within one hour of their collection and was assessed for volume, appearance, liquefaction, concentration, motility, morphology, viability and presence of pus cells. The descriptive statistical analysis was done.

## RESULTS

During the year 2017-2018, a total of 196 couple attended Gynecological OPD for infertility problems and consecutively entered in our study. Out of them three couple did not turn up with investigations report and seven of male partners refused to go for investigation. So, only 186 cases were analyzed in result. Mean age of the male was 28.3±5 years. Majority 130 (69.8%) had duration of infertility ≤5 years followed by 5-10 years 40 (21.2%) and only 17 (9.1%) had ≥10 years. Majority had secondary infertility. Different risk factors like (Mumps, diabetes, heat exposure, chemicals, Gulf country work etc) have been found to affect semen pattern and parameters adversely in our study. The numbers of patients suffered with mumps in the past that involved testis and found to be infertile were 5. Similarly, the patients exposed to heat exposure, chemical exposure, involved in smoking, consuming alcohol, suffering with diabetes mellitus, having hydrocele, varicocele and working in Gulf countries with infertility were 9, 14, 14,18,9,6,2,5 and 86 in our study as shown (Table 1). The most striking fact of this study is the maximum number of patients with infertility was from group of patients, who were working abroad Gulf countries.

**Table 1:** Distribution of risk factors for Infertility (n=186).

Main Risk Factors	n (%) of Partners
Golf worker	86 (46.2)
No risk Facors	35 (18.8)
Smoking	18 (9.6)
Chemicals Exposure	11 (5.9)
Heat exposure	9 (4.8)
Alcohol	9 (4.8)
Diabetes Mellitus	6 (3.2)
Mumps	5 (2.7)
Varicocele	5 (2.7)
Hydrocele	2 (1.1)
Total	186 (100)

**Figure 1:**
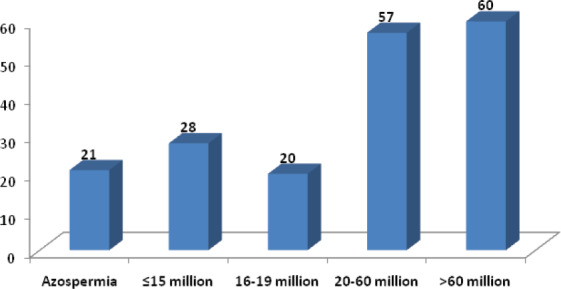
Distribution of Count of Spermatozoa of male partners.

Different parameters of the sperm were analyzed. These included volume, count, motility, morphology and pus cells. Culture of the semen of all the 186 patients was done. In our study, it was found that 101 (54.3%) patients had less than 2ml of semen volume. Similarly, the number of patients, who had semen volume 2-4 ml and more than 4 ml were 83 (44.6%) and 2 (1.1%) respectively as shown (Table 2). While analyzing the count, it was found that 21(11.2%) patients had azospermia in our study. Similarly, 60 (32.25%) patients were having more than 60 million sperm per ml as shown (Figure 1).

**Table 2:** Distribution of different characteristics of semen of male partners (n = 186).

Characteristics of semen	n (%) of male partners
Morphology of spermatozoa	
Normal	135 (72.5)
Abnormal	51 (27.4)
Number of pus cells/hpf	
>5	133 (71.5)
Absent	53 (28.5)
Volume of semen	
<2 ml	101 (54.3)
2-4 ml	83 (44.6)
>4 ml	2 (1.1)

**Figure 2:**
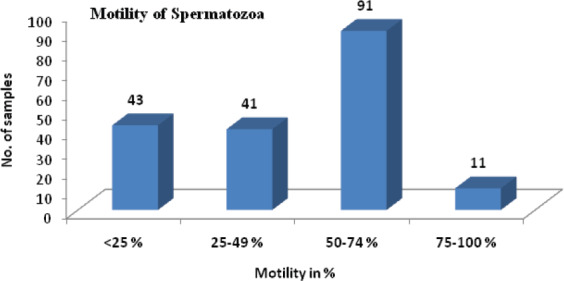
Distribution of motility of spermatozoa of male partners.

Our study found that 135 (72.5%) males had normal spermatozoa morphology and the rest were found to be abnormal as shown (Table 1). Forty three (23.11%) patients had less than 25% motile spermatozoa and only 11(5.91%) patients had 75-100% motile spermatozoa as shown (Figure 2). In 133 (71.5%) semen samples had more than 5 pus cells per high power field as shown (Table 1).

**Figure 3:**
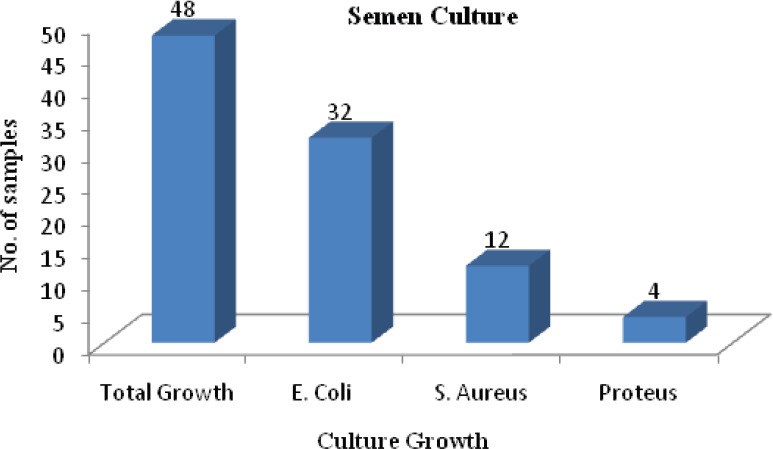
Distribution of growth of organism in semen sample of male partners.

Semen was also looked for the presence of organism by culture of the all samples. Forty eight (25.8%) semen samples showed the growth of organism in which E. Coli was isolated in 32 (17.2%) cases, S. Aureus in 12(6.45%) and Proteus in 4 (2.15%) as shown (Figure 3).

## DISCUSSION

During the analysis of the 186 male partners of infertile couples, we found the average age is 28±5 years which are similar with the study done in India.^8^ Majority of our patients had secondary infertility and sought medical help earlier than 5 years this may be because of the family pressure to have next child earlier, want to have next child when female is still young a, need of a friend to previous child and thinking that too much age gap between children makes couple difficult to care them properly. We found many risk factors like chronic smoking, alcohol abuse, diabetes, chemical exposure and other surgical interventions affecting the sperm quality and parameters. Above factors may adversely affect male fertility and surgical interventions may damage vas deference and vascular supply to the testis. Chronic smokers have high risk of infertility and toxins in tobacco smoke may kill sperms.^9,10^ Nine patients some from automobile garage who were mechanics and helpers, others were drivers who were constantly exposed to the heat coming from machines and vehicle engine respectively attended our clinic. We presume that this constant heat exposure to testis in association with other factors contributed to abnormal semen parameters in this group of patients. Many studies have shown that a 1-1.5°C increase in scrotal temperature leads to either impaired sperm production or abnormal sperm morphology.^11,12^ Epidemiological studies have revealed that more and more infertile men suffer from acute or chronic inflammation like mumps and chronic chemical exposure of the genitourinary tract, which often occurs without any symptoms.^13^ Chronic alcohol consumption causes sperm parameter abnormalities.^14^ It is very surprising that majority of the couple who sought medical help for infertility had their male partner worked in Gulf countries in the past. There may be multiple reasons behind this. The risk factors like severe stress, effect of climate change, sleeplessness, shift in working hours, exposure to high ambient and work place temperature, long sexual abstinence, adverse housing and work place situation and many others.

These factors, in susceptible person, may induce or precipitate organic and functional illnesses on top of the high temperature. The high temperature alone or in association with other risk factors may induce sperm parameter abnormalities which in turn lead to infertility. These issues should be addressed with large prospective studies involving migrant workers alone.

In analyzing the semen parameters, Eleven percent of our patients have azoospermia, 55% of the patients have sperm counts less than 60 million per ml. Absent and low sperm count is associated with male infertility.^15^ The sperm counts of male are declined with time.^16^ Forty five percent of our patients have sperm motility of less than 50%. The World Health Organization (WHO).^17^ Standards of normal sperm indicate that motility value should be greater than or equal to 50% with forward progression within 60 minutes of ejaculation. Low sperm motility is associated with infertility.^15^ Nearly one third of the males have abnormal sperm morphology which may have negative impact on fertility.

## CONCLUSIONS

To conclude with, multiple risk factors alone or in combination act to cause abnormal sperm parameters. One of our prominent finding is excessive exposure to heat in Gulf migrant workers that lead to abnormal sperm parameters. We found that abnormal semen analysis is the significant contributor to male infertility.

## Conflict of Interest

**None.**
